# Reinforcement Learning of Targeted Movement in a Spiking Neuronal Model of Motor Cortex

**DOI:** 10.1371/journal.pone.0047251

**Published:** 2012-10-19

**Authors:** George L. Chadderdon, Samuel A. Neymotin, Cliff C. Kerr, William W. Lytton

**Affiliations:** 1 Department of Physiology and Pharmacology, State University New York Downstate, Brooklyn, New York, United States of America; 2 Department of Neurobiology, Yale University School of Medicine, New Haven, Connecticut, United States of America; 3 School of Physics, University of Sydney, Sydney, Australia; 4 Department of Neurology, State University New York Downstate, Brooklyn, New York, United States of America; 5 Kings County Hospital, Brooklyn, New York, United States of America; Georgia State University, United States of America

## Abstract

Sensorimotor control has traditionally been considered from a control theory perspective, without relation to neurobiology. In contrast, here we utilized a spiking-neuron model of motor cortex and trained it to perform a simple movement task, which consisted of rotating a single-joint “forearm” to a target. Learning was based on a reinforcement mechanism analogous to that of the dopamine system. This provided a global reward or punishment signal in response to decreasing or increasing distance from hand to target, respectively. Output was partially driven by Poisson motor babbling, creating stochastic movements that could then be shaped by learning. The virtual forearm consisted of a single segment rotated around an elbow joint, controlled by flexor and extensor muscles. The model consisted of 144 excitatory and 64 inhibitory event-based neurons, each with AMPA, NMDA, and GABA synapses. Proprioceptive cell input to this model encoded the 2 muscle lengths. Plasticity was only enabled in feedforward connections between input and output excitatory units, using spike-timing-dependent eligibility traces for synaptic credit or blame assignment. Learning resulted from a global 3-valued signal: reward (+1), no learning (0), or punishment (−1), corresponding to phasic increases, lack of change, or phasic decreases of dopaminergic cell firing, respectively. Successful learning only occurred when both reward and punishment were enabled. In this case, 5 target angles were learned successfully within 180 s of simulation time, with a median error of 8 degrees. Motor babbling allowed exploratory learning, but decreased the stability of the learned behavior, since the hand continued moving after reaching the target. Our model demonstrated that a global reinforcement signal, coupled with eligibility traces for synaptic plasticity, can train a spiking sensorimotor network to perform goal-directed motor behavior.

## Introduction

Sensorimotor mappings, for example between proprioceptive input and motor output, are the basis for directed behavior, including foraging, locomotion, and object manipulation. Artificial neural networks, generally based on continuous unit states, have used a variety of learning algorithms to learn these mappings; examples include backpropagation [1], [2], self-organizing maps [3], and temporal difference learning [4].

Artificial neural network models, as well as lumped control theory models, use processing units with continuous outputs which encode continuous rates or probabilities of firing. By contrast, recent models have begun to look more closely at biomimetic mechanisms by using spiking models for dynamics and spike-timing-dependent plasticity (STDP) for learning [5]–[12]. Spiking units offer the advantage of allowing us to explore multiple methods of neural encoding that are absent from continuous unit models. These include exposing possible roles of synchrony in perceptual feature binding and response selection [13], wave-front encoding [14], [15], and other time-based codes. Physiologically, the degree of input spike synchrony is a major determinant of motor neuron activation [16].

Sensorimotor mappings can be thought of as stimulus-response mappings, suggesting reinforcement learning (RL) as a mechanism for learning. The essence of this learning mechanism was summarized over 100 years ago in Thorndike’s Law of Effect: stimulus-response mappings are strengthened by global reward and weakened by global punishment [17]. RL methods [18], including temporal-difference learning [4], have been used extensively in machine learning and offer an advantage over teacher-supervised learning methods in that they do not require a known desired output representation to match against the model’s current (behavioral) output. However, unlike unsupervised learning methods, they do offer some feedback regarding fitness of the behavior. A further framework for explaining motor RL is the perception-action-reward cycle [19]. The learning system is divided into an *actor*, mapping perceptions to actions (P to A), and a *critic* providing reward and punishment feedback to the actor [8], [20], [21]. To utilize this scheme, the naive actor must produce some actions. This is the role of *motor babble* [20], [22], [23], produced in our model via noise.

One challenge in the learning of actor/critic RL systems is the *distal reward* or *credit assignment problem* [7]: reinforcers are delivered after the behavior is complete, after synaptic and neuronal activations leading up to the output are no longer active. A synaptic eligibility trace can used to solve this problem: synapses are tagged to receive a credit or blame signal that arrives later [8]. Synapses tagged with eligibility traces, possibly mediated by transient phosphorylations [24] or dendritic Ca^2+^ currents [25], [26], may be reinforced by the global reinforcement signals mediated by phasic reward bursts [27], [28] and punisher dips [29] of dopamine cell firing from ventral tegmental area (VTA) projecting to cortical areas [30]–[34].

In this paper, we simulated a potential mechanism for the learning of sensorimotor mappings, using a biologically-inspired computational model consisting of spiking neuronal units whose synaptic weights are trained via global reward and punisher signals. This architecture was able to perform a stationary targeting task as long as both reward and punishment signals were present during the learning. Stable proprioceptive-to-motor command mappings mediated performance of the task.

## Methods

### Neuron Model

Individual neurons were modeled as event-driven, rule-based dynamical units with many of the key features found in real neurons, including adaptation, bursting, depolarization blockade, and voltage-sensitive NMDA conductance [35]–[40]. Event-driven processing provides a faster alternative to network integration: a presynaptic spike is an event that arrives after a delay at postsynaptic cells; this arrival is then a subsequent event that triggers further processing in the postsynaptic cells. Cells were parameterized as excitatory (E), fast-spiking inhibitory (I), and low-threshold-spiking inhibitory (IL; Table 1). Each cell had a membrane voltage state variable (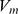), with a baseline value determined by a resting membrane potential parameter (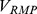). After synaptic input events, if 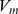 crossed spiking threshold (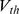), the cell would fire an action potential and enter an absolute refractory period, lasting 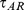. After an action potential, an after-hyperpolarization voltage state variable (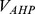) was increased by a fixed amount 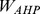 and then 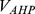 was subtracted from 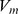. Then 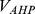 decayed exponentially (with time constant 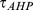) to 0. To simulate voltage blockade, a cell could not fire if 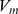 surpassed the blockade voltage (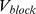). Relative refractory period was simulated after an action potential by increasing the firing threshold 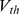 by 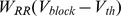, where 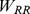 was a unitless weight parameter. 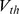 then decayed exponentially to its baseline value with time-constant 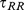.

**Table 1:** Parameters of the neuron model for each major population type.

Cell type	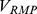 (mV)	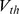 (mV)	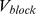 (mV)	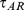 (ms)	*W_RR_*	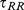 (ms)	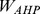 (mV)	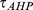 (ms)
Excitatory	–65	–40	–25	5	0.75	8.0	1.0	400
Inhibitory	–63	–40	–10	2.5	0.25	1.5	0.5	50
Low-threshold	–65	–47	–10	2.5	0.25	1.5	0.5	50

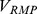
 = resting membrane potential; 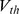 = threshold voltage; 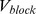 = blockade voltage; 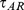 = absolute refractory time constant; 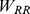 = relative refractory weight; 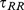 = relative refractory time constant; 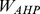 = after-hyperpolarization increment; 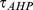 = after-hyperpolarization time constant.

In addition to the intrinsic membrane voltage state variable, each cell had four additional voltage state variables 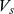 corresponding to synaptic input. These represent AMPA, NMDA, and somatic and dendritic GABA_A_ synapses. At the times of input events, synaptic weights were updated by step-wise changes in 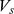, which were then added to the cell’s overall membrane voltage 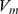. To allow for dependence on 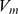, synaptic inputs changed 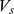 by 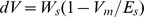, where 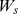 is the synaptic weight and 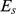 is the reversal potential relative to 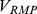. The following values were used for the reversal potential 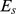: AMPA, 65 mV; NMDA, 90 mV; and GABA_A_, –15 mV. After synaptic input events, the synapse voltages 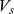 decay exponentially toward 0 with time constants 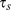. The following values were used for 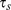: AMPA, 20 ms; NMDA, 300 ms; somatic GABA_A_, 10 ms; and dendritic GABA_A_, 20 ms. The delays between inputs to dendritic synapses (AMPA, NMDA, dendritic GABA_A_) and their effects on somatic voltage were selected from a uniform distribution ranging between 3–5 ms, while the delays for somatic synapses (somatic GABA_A_) were selected from a uniform distribution ranging from 1.8–2.2 ms. Synaptic weights were fixed between a given set of populations except for those involved in learning (described below).

### System Design

**Figure 1:**
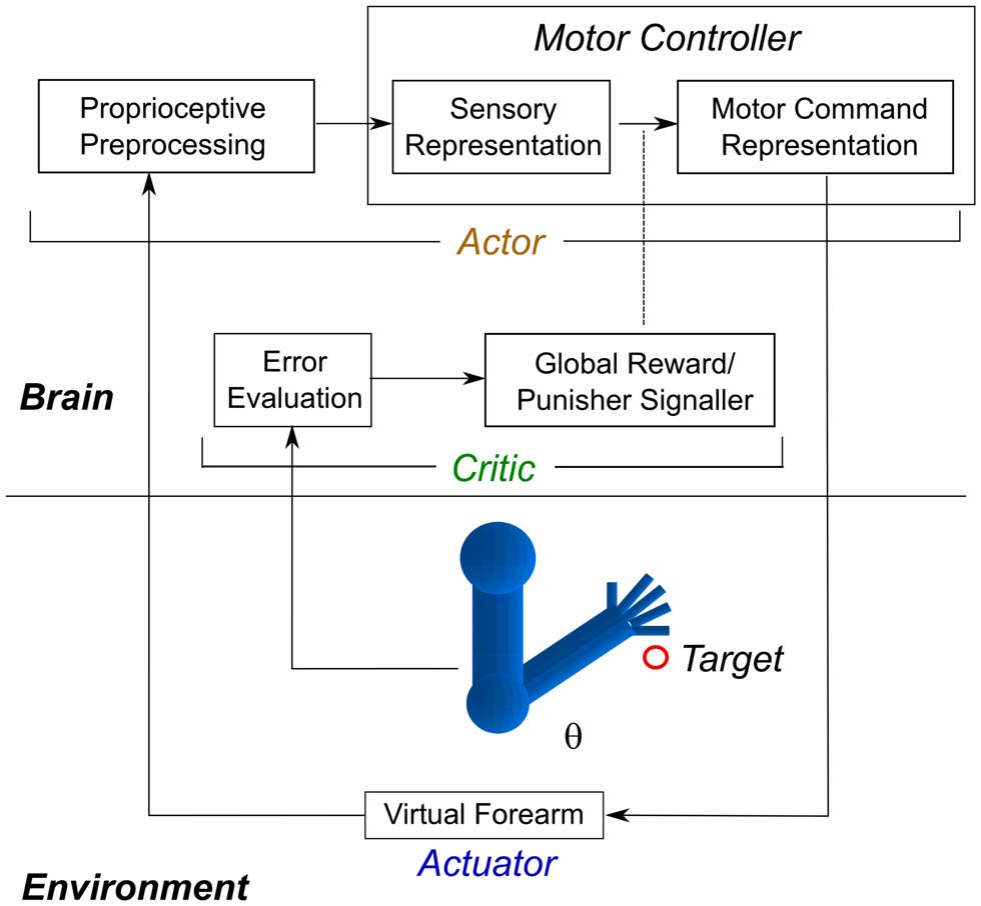
Overview of model. A virtual forearm with joint angle 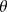, controlled by 1 flexor and 1 extensor muscle, is trained to align to a target. A *proprioceptive preprocessing* block translates muscle lengths into an arm configuration representation. Plasticity is restricted to the mapping between *sensory representation* and *motor command representation* units (dashed oval). Motor units drive the muscles to change the joint angle. The *Actor* (above) is trained by the *Critic* which evaluates error and provides a global reward or punishment signal.

The network system, shown in Fig. 1, consisted of (1) a simple one-joint “forearm,” with flexor and extensor muscles; (2) proprioceptive neurons, each tuned to fire at a specific joint angle; (3) sensory cells, which received spiking input from the proprioceptive cells; (4) motor command cells, which received spiking input from sensory cells and sent elbow rotation commands to the muscles; and (5) a reinforcement learning critic, which evaluated the change of hand-to-target visual error and sent a global reward or punisher training signal to the plastic synapses. The proprioceptive, sensory, and motor neurons were implemented using the model described above; further details on the system are provided below.

Input to the sensory cells was provided by 48 proprioceptive (P) cells, representing muscle lengths in 2 groups (flexor- and extensor-associated). Each was tuned to produce bursting approaching 100 Hz over a narrow range of adjacent, non-overlapping lengths. The cortical network consisted of both sensory and motor cell populations. The sensory (S) population included 96 excitatory sensory cells (ES cells), 22 fast spiking sensory interneurons (IS), and 10 low-threshold spiking sensory interneurons (ILS); similarly, the motor (M) network had 48 EM, 22 IM, and 10 ILM cells. The EM population was divided into two 24-cell subpopulations dedicated to extension and flexion, which projected to the extensor and flexor muscles, respectively.

Cells were connected probabilistically with connection densities and initial synaptic weights varying depending on pre- and post-synaptic cell types (Table 2). In addition to spikes generated by cells in the model, subthreshold Poisson-distributed spike inputs to each synapse of all units except the P and ES units were used to provide ongoing activity and babble (Table 3).

**Table 2:** Area interconnection probabilities and starting weight.

Presynaptic type	Postsynaptic type	Connection probability	Synaptic weight
P	ES	0.10	8.77
ES	IS	0.43	1.90
ES	ILS	0.51	0.95
ES	EM	0.08	5.28*
IS	ES	0.44	4.50
IS	IS	0.62	4.50
IS	ILS	0.34	4.50
ILS	ES	0.35	1.25
ILS	IS	0.53	2.25
ILS	ILS	0.09	4.50
EM	IM	0.43	1.90
EM	ILM	0.51	0.95
IM	EM	0.44	4.50
IM	IM	0.62	4.50
IM	ILM	0.34	4.50
ILM	EM	0.35	1.25
ILM	IM	0.53	2.25
ILM	ILM	0.09	4.50

* shows plastic connections, for which the initial weight is listed.

**Table 3:** Noise stimulation to synapses of the different cell types.

Cell type	Synapse type	Synaptic weight	Average rate (Hz)
IS	AMPA	4.13	300
IS	NMDA	1.50	50
IS	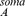 GABA	1.88	125
IS	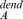 GABA	1.88	125
ILS	AMPA	3.00	300
ILS	NMDA	0.38	50
ILS	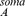 GABA	1.88	125
ILS	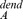 GABA	1.88	125
EM	AMPA	3.94	300
EM	NMDA	0.75	50
EM	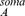 GABA	1.88	125
EM	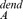 GABA	1.88	125
IM	AMPA	4.13	300
IM	NMDA	1.50	50
IM	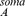 GABA	1.88	125
IM	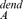 GABA	1.88	125
ILM	AMPA	3.00	300
ILM	NMDA	0.38	50
ILM	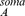 GABA	1.88	125
ILM	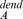 GABA	1.88	125

The virtual forearm consisted of a single segment of length 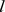 with a joint angle 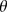 that was allowed to vary from 0° (arm straight) to 135° (fully flexed). An extensor and flexor muscle (lengths 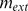 and 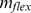) always reflected the current joint angle according to the following relationship:
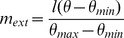
(1)

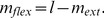
(2)


Arm position updates were provided at 50 ms intervals, based on extensor and flexor EM spike counts integrated from a 40 ms window that began 90 ms prior to update time (50 ms network-to-muscle propagation delay). The angle change 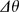 for the joint was the difference between the flexor and extensor EM spike counts during the prior interval, with each spike causing a 1° rotation. P drive activity updated after an additional 25 ms delay which represented peripheral and subcortical processing. Reinforcement occurred every 50 ms with calculation of hand-to-target error. The target remained stationary during the simulation.

The RL algorithm implemented Thorndike’s Law of Effect using global reward and punishment signals [17]. The network is the *Actor*. Feedforward ES 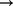 EM AMPA weights were trained to implement the learned sensorimotor mappings. The *Critic*, a global reinforcement signal, was driven by the first derivative of error between position and target during 2 successive time points (reward for decrease; punishment for increase). As in [7], we used a spike-timing-dependent rule to trigger eligibility traces to solve the credit assignment problem. The eligibility traces were binary-stated, turning on for a synapse when a postsynaptic spike followed a presynaptic within a time window of 100 ms; eligibility ceased after 100 ms. When reward or punishment was delivered, eligibility-tagged synapses were potentiated (long-term potentiation LTP), or depressed (long-term depression LTD), correspondingly.

Weights 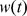 were updated utilizing weight scale factors, 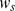:



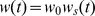
.
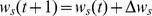


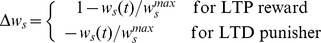
where 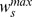 (5 in all simulations) is maximum weight scale factor, and 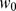 is the initial synaptic weight. 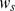 is initialized to 1.0 for all synapses and varies between 0 and 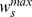.

The model was implemented in NEURON 7.2 [41] for Linux and is available on ModelDB (https://senselab.med.yale.edu/modeldb). One minute of simulated time took approximately 80 s of CPU time on an Intel XEON 2.27 GHz CPU.

## Results

**Figure 2:**
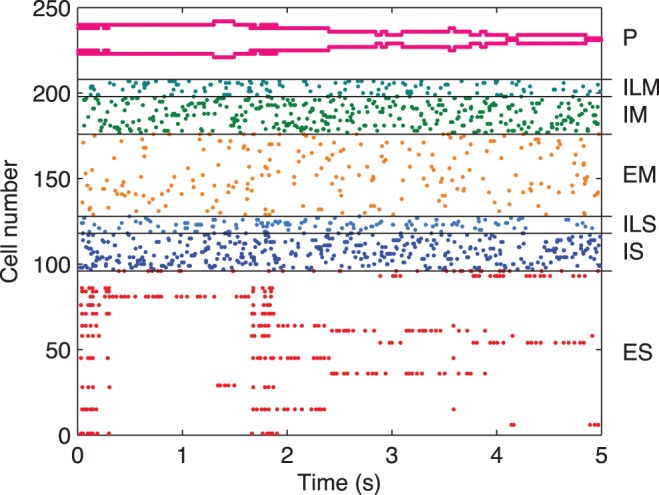
Baseline spiking before learning. Raster of spikes from individual cells: ES, excitatory sensory units; IS, fast-spiking inhibitory sensory units; ILS, low-threshold inhibitory sensory units; EM, excitatory motor units; IM, fast-spiking inhibitory motor units; ILM, low-threshold inhibitory motor units; and P, proprioceptive input units.

Average spiking rates (in Hz) were: P, 1.9; ES, 0.4; IS, 4.4; ILS, 2.9; EM, 0.5; IM, 4.3; and ILM, 3.1, in the absence of learning and the presence of babble noise (Fig. 2). Inhibitory cells fired faster than excitatory cells, consistent with observed rates in cortex. The top units in Fig. 2 represent the proprioceptive (P) inputs from the flexor and extensor muscles. Each of the 2 muscles stimulates 1 or 2 of the P cells to fire, with the particular cells depending on current muscle length.

**Figure 3:**
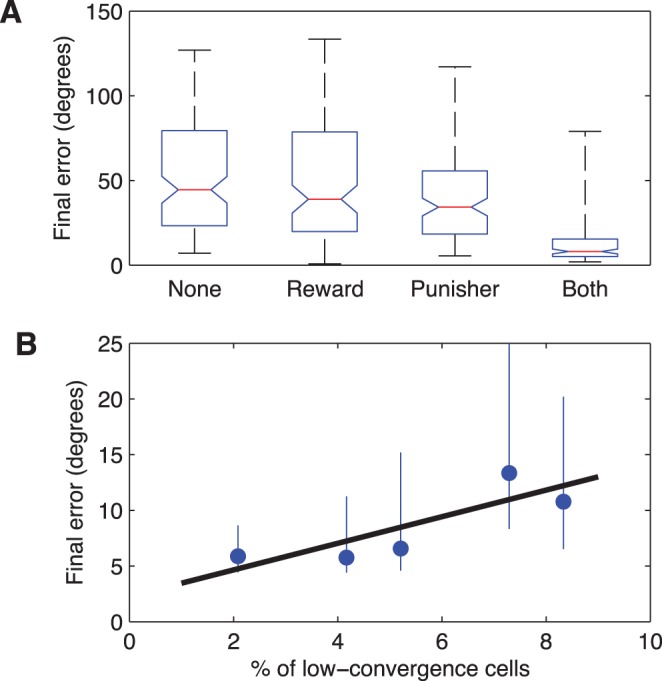
Performance across learning conditions and random wiring seeds. A. End-of-trial errors under different learning conditions: no learning, reward-only, punisher-only, reward-and-punisher; 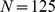 for each: 5 target angles (0°, 35°, 75°, 105°, 135°)×5 random wirings×5 random babble noise inputs. B. Learning performance as a function of the percentage of EM cells that have low-convergence, defined as having fewer than five afferent inputs. The final error appears to be strongly correlated with the proportion of low-convergence cells (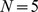; Pearson’s 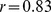, 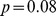).

We ran 500 simulations (4 learning modes: no learning, reward-only, punisher-only, reward-and-punisher×5 target angles: 0°, 35°, 75°, 105°, 135°×5 wiring random seeds×5 babbling noise input random seeds) to assess performance of the model in learning to reach for a single target. For each trial, final error was measured by the average absolute value angle difference between hand and target in the last 20 s of the 200 s trials. A Shapiro-Wilk test on all final errors for the reward-and-punisher condition found that the null hypothesis of normality could be rejected (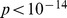), so non-parametric statistics have been used throughout. The reward-and-punisher learning algorithm showed clear superiority over reward-only or punisher-only methods (Fig. 3A; 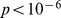, Kruskal-Wallis test). Final error was much less with both reward and punishment (median = 8.07, IQR = 5.10–15.23) than with the other cases (median = 38.96, IQR = 19.85–78.53 for the reward-only condition). Therefore, reward-and-punisher learning was used for all of the additional studies.

**Figure 4:**
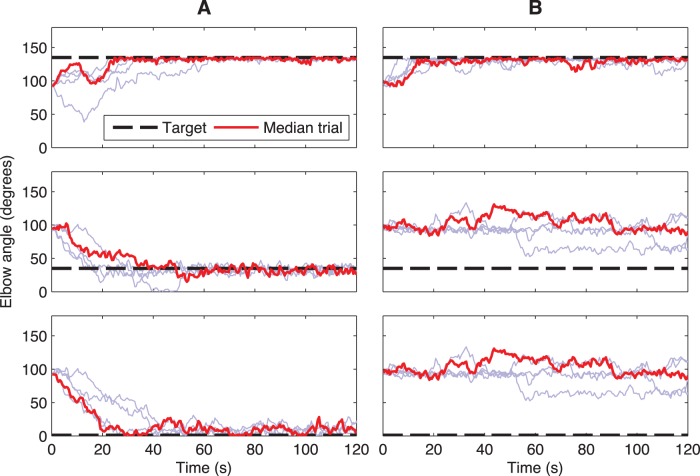
Target learning for two model wirings. Columns A and B show different wiring seeds, while each panel shows 5 different babble input random seeds with median final performance in red (calculated over 100–200 s; only 0–120 s shown).

Learning with this algorithm was successful with arbitrary choice of random babble inputs. However, some randomly chosen wirings produced networks that would not learn adequately across the set of targets (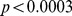 difference for wirings; Kruskal-Wallis test with Bonferroni correction). The difference across different network architectures was explained by noting that poorly performing networks had a relatively high proportion of poorly connected EM neurons, defined as cells with fewer than five inputs from sensory cells (Fig. 3B). These neurons would not receive adequate drive and would therefore contribute less to the dynamics. Conversely, networks with more consistent numbers of inputs per cell were more flexible and thus better at learning. In addition, the wirings that produced unevenly-performing (across targets) networks often appeared to have a strong innate bias towards flexion or extension. Fig. 4 compares a wiring that produced good learning across all 5 targets (3 shown; Fig. 4A) compared to another wiring that would only learn the 2 most flexed targets (1 shown; Fig. 4B). Note that all the random babble activations in Fig. 4B produced networks that learned the flexion position (135°) and did not learn any other position, save 105° (not shown). Furthermore, these networks learned flexion more quickly than any of the networks of Fig. 4A.

The successful performance seen in Fig. 4A might have depended on metastable learning: learning due solely to ongoing reward and punishment feedback changing the proprioceptive-to-motor mapping in an *ad hoc* fashion, as opposed to a stable mapping being learned to guide the arm in the correct direction. We therefore tested for stability by assessing performance with learning turned off after training. Performance remained stable with median final error of 6.8° (IQR = 4.1–13.0; *N* = 125 simulations), statistically unchanged from the final error with learning turned on. Stable learning suggested the development of an attractor around the target that was sufficiently deep to compensate for deviations produced by ongoing babble input.

**Figure 5:**
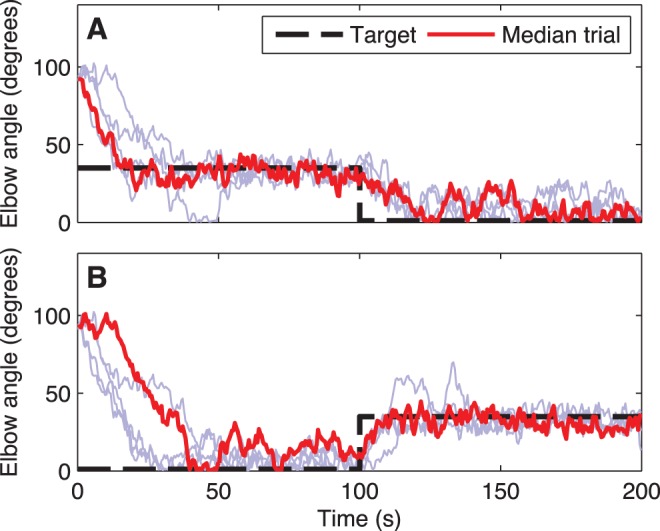
Serial target learning. A. 35° 0°. B. 0° 35°. (blue: 4 babble traces; red: median trial; wiring from Seed 1 of Fig. 4.).

In order to assess learning algorithm adaptability to altered environmental circumstances, we switched targets after training (targetswitch). We performed a Wilcoxon sign-rank test to compare performance on the first and second target positions and found that there was no significant difference (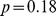, 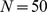). Likewise, when we performed a Wilcoxon rank-sum statistic test comparing performance on the first target sequence (Fig. 5A) vs. the second (Fig. 5B), we found no significant difference (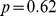, 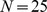). This suggests that target switching performance is independent of target ordering or place in the sequence. The new target was generally learned rapidly, within 25 s after the switch. Those wirings that could not learn the separate single target trials for both first and second target could also not learn target switching between the two.

**Figure 6:**
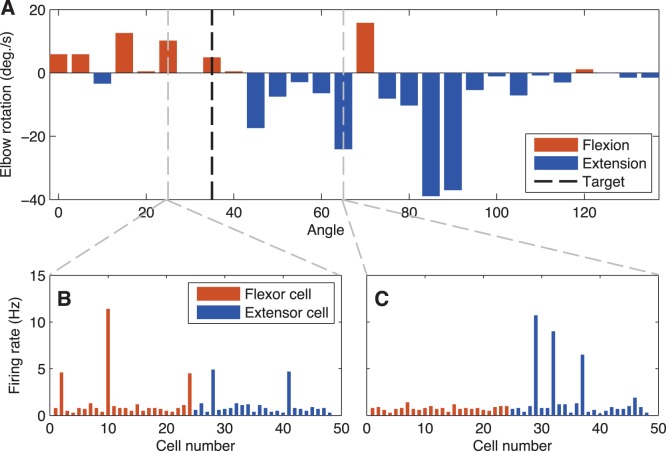
Learned EM activation for target of 35°. A. Rotation command (differences of flexor and extensor EM rates) as a function of angle. Example firing rates for individual cells are shown at two representative arm angles: 25° (B, extended relative to target) and 65° (C, relatively flexed).

Plasticity was confined to ES→EM weights. Reward-only and punisher-only algorithms each only allowed change in 1 direction and thus produced monotonic alterations in weights. The reward-and-punisher algorithm produced a net increase in weights, but allowed weights to balance in a way not possible with only one learning direction. Separate populations of EM cells drove extension and flexion of the virtual forearm, with overall arm motion being determined by the difference between the aggregate subpopulation firing rates. To understand the net effect of learning, we examined the activity produced at each arm angles at increments of 5°. Fig. 6A shows the rotation commands that are the differences of flexor (red) and extensor (blue) EM cell aggregate firing rates for post-learning. The black vertical line indicates the target the model was trained to reach for (35°). The dotted vertical line at 25° indicates a condition where the arm angle is less than the target, and the dotted line at 65° indicates a condition where the arm angle is greater than the target. Fig. 6B shows the firing of the individual EM cells under the over-extended example (25°). Both flexion and extension EM cells learn to burst here in response to 25°, but the net firing of the flexion cells (red) wins. Fig. 6C shows the firing of the individual EM cells under the over-flexed example (65°). Here, only extension cells effectively learn to burst in response to 65°, making extension (blue) the clear winner. Learning generally caused certain cells to burst at rates up to 12 Hz in response to particular detected angles. With some notable exceptions (e.g. 10° and 70°), Fig. 6A indicates that flexion wins, as is desired, when the arm angle is less than the target, and extension wins when the angle is greater. The aggregate learning effect is an attractor around the target at 35°.

## Discussion

We have created a model of motor reinforcement learning for a simple target reaching task for a virtual forearm, using spiking units whose weights are trained using a reinforcement learning algorithm. Baseline spiking rates were adjusted to be comparable with rates observed in cortical tissue (Fig. 2). Both reward and punishment together were essential for learning the task (Fig. 3A). The model was able to learn a stable attractor for the target, not merely relying on metastable, ad hoc learning for performance. The model was not only able to learn under different initial target training situations, but was able to relearn (Fig. 5). Reinforcement learning worked by shaping the collective activity of the EM cells through synaptic plasticity in the ES to EM projection (Fig. 6). This plasticity shaped EM responses to the proper mixture of ES activations to produce the desired behavior. We note that the simplicity of our one degree-of-freedom task, compared to the complexities of limb movement, reduces the scope of what can be demonstrated in this model. In particular, the network did not need to obtain stable intermediate synaptic strengths, as would be required in higher-dimensional tasks.

We predict that learning of a motor task will require both reward and punishment signals, hence both LTP and LTD in motor cortex, regulated through differential dopaminergic signaling. However, there are many additional synaptic influences in neocortex that were not included in this model. Adaptation in cell firing rate based on global synaptic input (homeostasis) or interactions between the strength of synapses (normalization) could provide alternative ways of achieving synaptic decrementation without punishment/LTD. All of frontal cortex, including M1, is innervated extensively by dopaminergic projections from the ventral tegmental area which could provide regulation of plasticity [30]–[32]. There is evidence that increased dopamine concentration leads to synaptic LTP and that decreased dopamine concentration leads to synaptic LTD mediated via action of D1-family receptors [42], [43]. We therefore predict that dopaminergic innervation of M1 from the VTA would be required for learning, and that antagonism of D1 would be likely to impair acquiring of motor tasks. Various kinds of learning are impaired by disruption of dopaminergic pathways [44]–[48]. Parkinson disease patients, who have damage to midbrain dopaminergic nuclei, including VTA [49], have deficits reward-based learning [49]–[51].

Stable learned mappings can still permit rapid learning and unlearning due to shifting reinforcement conditions (Fig. 5). Similar metastable behavior is seen in the ongoing error corrections in adult bird-song production, which relies on sensorimotor integration [52]. Shifting reinforcement conditions are a typical feature of an animal’s environment. Areas of the habitat once rich in food may become depleted, or once-safe areas may later be occupied by predators, making the capability for rapid unlearning and relearning of reward and punishment conditions important for survival.

Babble noise in a motor system is likely to be important in the exploration needed to drive successful animal reinforcement learning. Random motor activity provides the variation required by selection. Movements or programs can then be reinforced, consistent with a selective hypothesis [53]. This interaction of babble and learning has been most clearly demonstrated in the variability of Bengalese finch bird-song. In this species, the lateral magnocellular nucleus of the anterior nidopallium (LMAN), a part of the basal-ganglia forebrain circuit, projects to pre-motor areas which activate song production. LMAN provides a source of variability which is required for song-learning to take place via a process of random exploration and learning [54], [55]. In primates, exploratory behavior has been associated with activity in anterior cingulate cortex (ACC) [56], [57] and frontopolar cortex (BA 10) [58], [59], which is also connected to ACC [60]. It has been proposed that there may be a ventral striatal-to-cortical loop gating activity in one or both of these areas that mediates the onset and offset of motor babbling noise applied to the cortical actor [20].

In our present model, babble remained at the same level throughout learning. Babble thus interfered with the stability of learned mapping. This interference could be reduced by setting babble noise adaptively to reflect the current level of reward and punishment that the actor is receiving: high levels of punishment or low levels of reward should encourage babbling, whereas high levels of reward should discourage it [20]. This would reduce overall exploratory behavior, but allow it to be re-engaged during environmental change. Variability in the motor system should be maintained, yet be carefully regulated [55].
