# Correction: Reinforcement Learning of Targeted Movement in a Spiking Neuronal Model of Motor Cortex

**DOI:** 10.1371/annotation/f6185650-eb8d-430b-9410-d079c56cef7f

**Published:** 2013-04-26

**Authors:** George L. Chadderdon, Samuel A. Neymotin, Cliff C. Kerr, William W. Lytton

The copyright statement is incorrect. The correct copyright statement is:

© Chadderdon et al. This is an open-access article distributed under the terms of the Creative Commons Attribution License, which permits unrestricted use, distribution, and reproduction in any medium, provided the original author and source are credited.

Figure 1 is incorrect. The correct Figure 1 can be viewed here: 

**Figure:**
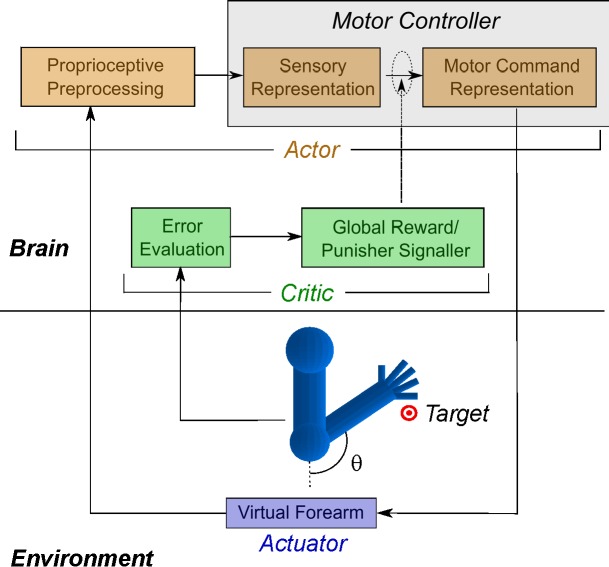


[^]

Table 3 was formatted incorrectly. The correct Table 3 can be viewed here: 

**Figure:**
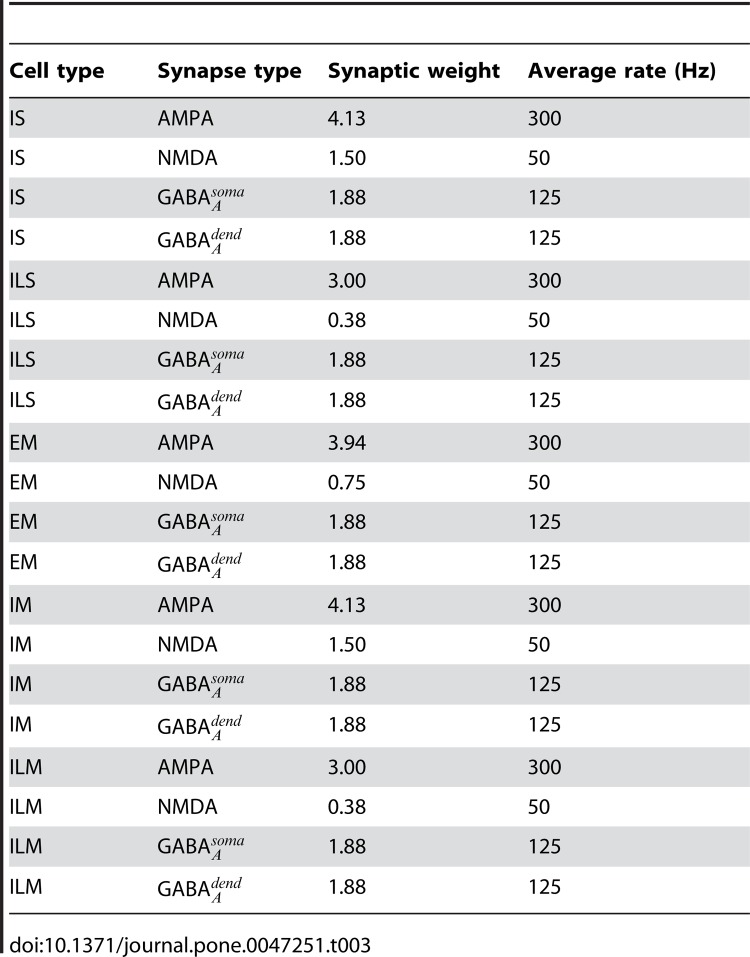


[^]

Two symbols were omitted in the Figure 5 legend. The correct legend and figure can be viewed here: 

**Figure:**
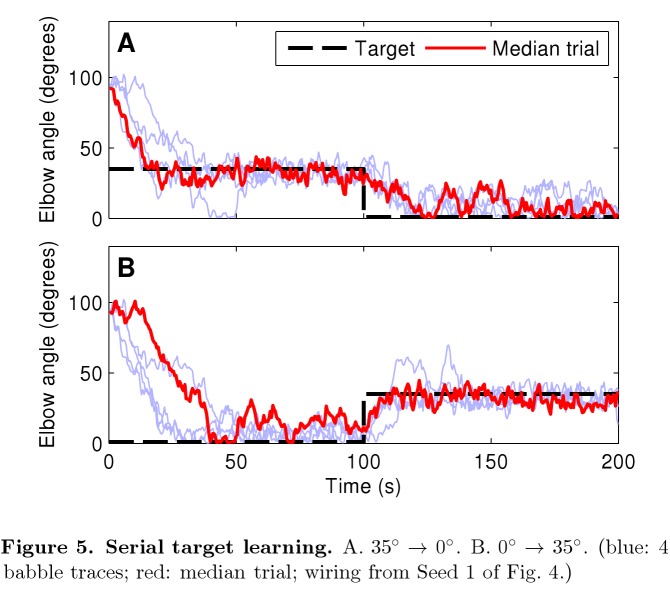


[^]

In the fifth paragraph of the results section, the first sentence should read:

In order to assess learning algorithm adaptability to altered environmental circumstances, we switched targets after training (Figure 5).

